# Mechanistic study of lncRNA UCA1 promoting growth and cisplatin resistance in lung adenocarcinoma

**DOI:** 10.1186/s12935-021-02207-0

**Published:** 2021-09-20

**Authors:** Jiali Fu, Jingjing Pan, Xiang Yang, Yan Zhang, Fanggui Shao, Jie Chen, Kate Huang, Yumin Wang

**Affiliations:** 1 Department of Laboratory Medicine, The First Affiliated Hospital of Wenzhou Medical University, Wenzhou, 325000 China; 2 Department of Intensive Care Unit, The First Affiliated Hospital of Wenzhou Medical University, Wenzhou, 325000 China; 3 Department of Pathology, The First Affiliated Hospital of Wenzhou Medical University, Wenzhou, 325000 China

**Keywords:** Cisplatin resistance, lncRNA, Lung adenocarcinoma, Tumor development, UCA1

## Abstract

**Aim:**

This study aimed to explore the mechanism of LncRNA urothelial carcinoma-associated 1 (UCA1) promoting cisplatin resistance in lung adenocarcinoma (LUAD).

**Method:**

The UCA1 expression level in LUAD cell lines was detected by reverse transcription‑quantitative polymerase chain reaction (RT‑qPCR). We overexpressed UCA1 in A549 cells and downregulated UCA1 in A549/DDP cells by the lentivirus‑mediated technique. Subsequently, in vitro, and in vivo functional experiments were performed to investigate the functional roles of UCA1 in the growth and metastasis of LUAD cell lines. Furthermore, RNA pulldown, mass spectrometry, and RNA immunoprecipitation technique were performed to analyze various downstream target factors regulated by UCA1.

**Results:**

The results revealed a higher UCA1 expression level in A549/DDP cells and LUAD tissues than in A549 cells and adjacent cancer tissues. UCA1 expression was significantly associated with distant metastasis, clinical stage, and survival time of patients with LUAD. UCA1 overexpression significantly increased the proliferation, invasion, clone formation, and cisplatin resistance ability and enhanced the expression levels of proliferating cell nuclear antigen and excision repair cross-complementing gene 1 in A549 cells. However, these trends were mostly reversed after the knockdown of UCA1 in A549/DDP cells. Tumorigenic assays in nude mice showed that UCA1 knockdown significantly inhibited tumor growth and reduced cisplatin resistance. Enolase 1 was the RNA-binding protein (RBP) of UCA1.

**Conclusion:**

Based on the results, we concluded that UCA1 promoted LUAD progression and cisplatin resistance and hence could be a potential diagnostic marker and therapeutic target in patients with LUAD.

**Supplementary Information:**

The online version contains supplementary material available at 10.1186/s12935-021-02207-0.

## Introduction

Lung cancer was the second most diagnosed cancer and the leading cause of cancer death, with an estimated 2.2 million new cancer cases and 1.8 million deaths in 2020 [1]. The current 5-year survival rate of patients with lung cancer was lower than 20% due to the absence of validity for diagnosis in the early stage and the lack of effective therapies for advanced lung cancer, [2]. Lung adenocarcinoma (LUAD) was the most common histological type of non-small cell lung cancer (NSCLC).

Notably, platinum-based chemotherapy was still the first-line treatment for advanced NSCLC and an efficient method to improve the survival rate and life quality of patients [3]. Inevitably, platinum‐based drugs and targeted drugs, which achieved a significant effect at the beginning of treatment, had limitations due to the development of resistance with long-term use [4]. The mechanism of resistance to cisplatin (DDP) was extremely complex, involving multiple genes, and was currently thought to be achieved primarily through multiple mechanisms [5–7]. Despite many advances in genomic and proteomic studies, the mechanism of cisplatin resistance remained elusive.

Meanwhile, accumulating evidence indicated the involvement of long noncoding RNA (lncRNA) in cancer development, progression, and drug resistance [8–15]. LncRNA urothelial carcinoma-associated 1 (UCA1) was first reported by Wang [16]. UCA1 belongs to the human endogenous retrovirus H family, with a full length of 1439 bp and without protein translation. It was one of the most distinctive genes for bladder cancer [16].

Some reports were available regarding the role of UCA1 in chemoresistance. UCA1/miRNA204-5p/cAMP responsive element binding protein-1 (CREB1) axis [17], UCA1/miRNA-143/Fos-like antigen 2 (FOSL2) axis [18], and UCA1/miRNA-196a-5p/CREB axis [19] were involved in enhancing the chemoresistance of colorectal, ovarian, and bladder cancers. In NSCLC, Li et al. [20] discovered complementary binding sites between UCA1 and miRNA-495, and indicated that UCA1 promoted cisplatin resistance of NSCLC by regulating the miRNA-495/Nuclear factor-erythroid 2-related factor 2 (NRF2) axis as ceRNA. Liu et al. [21] found that epithelial–mesenchymal transition (EMT) related proteins had changed and LUAD cell lines restored the sensitivity of cisplatin after UCA1 knockdown, which meant that UCA1 promoted cisplatin resistance by participating in the EMT signaling pathway. However, the specific regulatory relationship between UCA1 and chemoresistance in NSCLC was still unclear.

In our previous experiments, differentially expressed lncRNAs between LUAD cisplatin-sensitive cell line A549 and LUAD cisplatin resistant cell line A549/DDP were screened using a lncRNA chip. We found that UCA1 was highly expressed in A549/DDP cells. Hence, we speculated that UCA1 played a vital role in the cisplatin resistance of LUAD. A mechanistic study of lncRNA UCA1 promoting cisplatin resistance in LUAD was also performed.

## Materials and methods

### Human LUAD tissue samples

The clinicopathological features and UCA1 expression level in 433 patients with LUAD were downloaded from The Cancer Genome Atlas (TCGA) database. The age of patients ranged from 33 to 88 years (median age 66 years), including 198 men and 235 women. UCA1 expression in LUAD and normal tissues, and the survival curve in the TCGA database were obtained through the analysis of the UALCAN website (http://ualcan.path.uab.edu/index.html) [22].

### Cell culture

A549 and A549/DDP cells were purchased from the Cell Bank of the Chinese Academy of Sciences (Shanghai, China). The cell lines were cultured in a Roswell Park Memorial Institute1640 (RPMI 1640, Thermo Fisher Scientific, MA, USA) medium containing 10% fetal bovine serum (FBS, Thermo Fisher Scientific) and maintained in an incubator at 37 °C, in the presence of 5% CO_2_ in a humidified atmosphere. The cell culture medium was changed every 2–3 days. When near confluence, the culture medium was discarded, and 2 mL of PBS (phosphate buffer saline) was added to rinse. The cells were digested using pancreatic enzymes, made into a single-cell suspension, and sub-cultured in a ratio of 1:3. For the culture of A549/DDP, 1 µg/mL cisplatin (Beyotime, Shanghai, China) was added to the RPMI1640 medium described earlier to maintain cisplatin resistance capability.

### Cisplatin sensitivity test

A single-cell suspension was prepared. The cells were seeded into 96‐well plates (4 × 10^3^ cells/well) and cultured overnight. The next day, the culture medium in the well was removed, and the cells were incubated with different concentrations of cisplatin (0, 1, 2, 4, and 8 µg/mL) for 48 h. Then, the culture medium in each well was replaced with 90 µL of RPMI 1640 and 10 µL of Cell Count Kit 8 (CCK8, Dojindo, Kumamoto, Japan) solution. After 1 h, the absorbance value was measured at 450 nm with a microplate reader (Tecan company, Mannedorf, Switzerland). Cell viability % = (*A*_*plus*_ − *A*_*blank*_)/(*A*_*0 plus drug*_ − *A*_*blank*_) × 100%. The half-maximal inhibitory cisplatin concentration (IC50) was calculated using a SPSS21.0 software profit regression model.

### Cell viability assay

A single-cell suspension was prepared. The cells were seeded into 96‐well plates (2 × 10^3^ cells/well) and the cisplatin-containing medium in the cisplatin treatment groups was replaced after the cells were attached completely. According to the IC50 value of cells, the cisplatin concentration of the medium in the UCA1 overexpression groups was 2 μg/mL, and that in the UCA1 knockdown groups was 4 μg/mL. The next day, the culture medium in the well was removed, and 100 μL of the culture medium containing 10% CCK8 was added to each well. The absorbance value was measured at 450 nm after 1 h. This procedure was repeated for 5 consecutive days.

### Cell migration and invasion assays

The migration and invasion assays were performed with 8.0-μm-pore inserts in a 24‑well plate. For the migration assay, 5 × 10^4^ cells and 100 μL of RPMI 1640 were added into the upper compartment of the Transwell inserts, and 200 μL of the culture medium containing 30% FBS was added into the lower compartment. The invasion assay was performed with Matrigel‑coated filters. In the cisplatin treatment groups, RPMI 1640 and the culture medium, containing 2 μg/mL cisplatin (UCA1 overexpression group) and 4 μg/mL cisplatin (UCA1 knockdown group), was added to the upper and lower chambers respectively. The cells were incubated for 24 h (migration assay) and 48 h (invasion assays). The migrated and invaded cells were fixed with methanol and stained with 0.1% (w/v) crystal violet. Finally, five fields were randomly taken under the microscope to perform cell counting and statistical analysis of results. Each experiment was performed three times.

### Colony formation assay

A total of 300 cells were seeded into 12-well plates. In the cisplatin treatment groups, a medium containing 2 μg/mL cisplatin (UCA1 overexpression group) and 4 μg/mL cisplatin (UCA1 knockdown group) was added. After 14 days of incubation, the cells were fixed with 4% paraformaldehyde for 15 min and stained with 0.1% (w/v) crystal violet for 15 min. The cell colonies (more than 50 cells) were counted, which indicated the ability of cell clone formation. The assay was conducted three independent times.

### Constructed lentivirus-mediated overexpression and shRNA vector

The overexpression vector targeting UCA1 as well as a negative control (NC, Genechem, Shanghai, China) were transfected into A549 cells. A549/DDP cells were transfected with an shRNA vector targeting UCA1 and shNC (Genechem). Transfection was performed by seeding 2 × 10^5^ cells into a six-well plate, and the medium was aspirated and incubated with a transfection complex after 24 h following the manufacturer’s protocol [multiplicity of infection (MOI) value was 10]. The A549/DDP and A549 cells were infected with lentivirus for 72 h and treated with 2 μg/mL puromycin to obtain cells transfected with the vector successfully. The transfection efficiency was detected by RT-qPCR. Detailed target sequences of shRNA and NC were as follows: CTCCTGGAAGCCACAAGATTA and TTCTCCGAACGTGTCACGT.

### Real-time RT-PCR

Total RNA from cells was isolated using TRIzol reagent (Thermo Fisher Scientific) and reverse-transcribed into cDNA using a PrimeScript RT Reagent Kit (TaKaRa, Dalian, China), following the manufacturer’s protocol. The expression levels of genes were normalized to the levels of the internal control β-actin by the 2^−ΔΔCT^ method. Quantitative polymerase chain reaction (PCR) assays were carried out on ABI 7500 (Thermo Fisher Scientific). The detailed primer sequences are shown in Table 1. A 10 μL PCR system contained 1 μL of cDNA template, 0.4 μL of 10 μM preserve primers, 0.4 μL of 10 μM forward primers, 5 μL of TaKaRa TB Green™ Premix Ex Taq™ II (TaKaRa), 0.2 μL of ROX II (TaKaRa), and 3 μL of ddH_2_O. PCR conditions were as follows: 30 s at 95 °C, followed by 40 cycles of 5 s at 95 °C, 34 s at 55 °C, and 15 s at 95 °C; and 1 cycle of 15 s at 95 °C, 1 min at 60 °C, and 15 s at 95 °C. The reactions were performed independently in triplicate.

**Table 1:** Detailed primer sequences for real-time RT-PCR

Gene	Primer sequences	
	Forward	Reverse
UCA1	ACGCTAACTGGCACCTTGTT	CTCCGGACTGCTTCAAGTGT
β-Actin	CCTGGCACCCAGCACAAT	GCTGATCCACATCTGCTGGAA
ENO1	ACCCAGTGGCTAGAAGTTCAC	CCATGGGCTGTGGGTTCTAA

### Western blot analysis

For harvesting the protein, the cells were washed twice with PBS and lysed with 100 μL of ice-cold 1× Radio Immunoprecipitation Assay (RIPA) lysis buffer (Beyotime) after growing to 80% density. For each sample, 20 μg protein was subjected to Sodium Dodecyl Sulfate Polyacrylamide Gel Electrophoresis (SDS‐PAGE) at 70 V for 40 min, and 120 V for 60 min and then transferred to a Poly Vinylidene Fluoride (PVDF) membrane (Millipore, MA, USA) at 300 mA for 60–120 min in an ice bath. The membrane was incubated with antibodies, including excision repair cross complementing gene 1 (ERCC1), proliferating cell nuclear antigen (PCNA), survivin, and β-actin (Proteintech, IL, USA), overnight at 4 °C. After washing with PBST, the PVDF membrane was incubated with secondary antibody (Beyotime) for 1 h at room temperature. An enhanced chemiluminescence (ECL) kit (Thermo Fisher Scientific) was used to visualize and analyze the expression of indicated proteins. Finally, the protein bands were scanned and analyzed in Alpha View software for gray values. The grayscale of targeted bands was normalized to the grayscale of β-actin, and the relative grayscale was analyzed using SPSS software.

### RNA pulldown and mass spectrometry

RNA pulldown assays were performed with a Pierce Magnetic RNA–Protein Pull-Down Kit (Thermo Fisher Scientific) following the manufacturer's protocol. The primer sequences of sense chain and antisense chain of lncRNA UCA1 are listed in Additional file 2: Table S1. Sense and antisense transcripts of UCA1 were labeled using Biotin RNA Labeling Mix (Roche Diagnostics, Mannheim, Germany). The labeled RNA was mixed with magnetic beads (Thermo Fisher Scientific). The mixture of RNA and magnetic beads was added to the cell lysate and incubated to form an RNA–protein complex. The supernatant was purified and analyzed by Western blot, silver staining, and protein mass spectrometry.

The raw Mass spectrometry/Mass spectrometry files of the mass spectrometer were submitted to ProteinPilot (https://sciex.com.cn/products/software/proteinpilot-software, version 4.5, SCIEX, Redwood City, California, USA) for data analysis. The parameters are set as follows: the instrument is TripleTOF 5600, the cysteine is modified with iodoacetamide; the biological modification is selected as the ID focus. For the identified protein results, certain filtration criteria were selected, and the peptide with an unsed score > 1.3 (more than 95% confidence) was considered a trusted peptide, retaining a protein containing at least one unique peptide.

### RNA immunoprecipitation

The RNA immunoprecipitation (RIP) assays were conducted using a Magna RIP RNA-Binding Protein Immunoprecipitation Kit (Millipore) following the manufacturer’s protocols. In brief, the cell extracts were mixed with protein A/G beads conjugated to an antibody against Enolase 1 (ENO1) or IgG (NC, Proteintech). Then, the precipitated RNAs were analyzed by qPCR.

### In vivo tumorigenic ability

A total of 24 BALB/c nude mice (male, 4 weeks) were purchased from the experimental animal center at Wenzhou Medical University (Wenzhou, China). The protocols of animal studies were approved by the Animal Experimental Ethical Inspection of Laboratory Animal Centre, Wenzhou Medical University (WYDW2020-0380). The mice were maintained and treated under specific pathogen-free (SPF) conditions and randomly divided into four groups (A549/DDP-Lenti-shNC, A549/DDP-Lenti-shUCA1, A549/DDP-Lenti-shNC + DDP, and A549/DDP-Lenti-shUCA1 + DDP). Further, 1 × 10^7^ cells were implanted into the right flank of mice via subcutaneous injection. Tumor sizes were calculated using a Vernier caliper as follows: tumor volume (mm^3^) = (*L* × *W*^2^)/2, where L = long axis and W = short axis. For the cisplatin treatment group, once the tumor size was 50 mm^3^, cisplatin (0.35 mg/kg) was injected intraperitoneally twice a week for 4 weeks. On the 38th day of the experiment, the mice were anesthetized (induction under 5% isoflurane, 1–2% continued isoflurane to maintain anesthesia), and anesthetic depth was confirmed by toe pinch. Euthanized animals were then subjected to cervical dislocation to ensure euthanasia, and excised xenograft tumors were weighed.

### Statistical analysis

Statistical analyses were performed using SPSS 21.0 software and the two-sample t-test. The clinical data were analyzed using the chi-square test or Fisher’s exact test. *P* values < 0.05 indicated statistically significant difference. NS meant not significant; *P < 0.05; **P < 0.01; ***P < 0.001.

## Result

### UCA1 was highly expressed in LUAD tissues and cells and was related to the prognosis

**Fig. 1:**
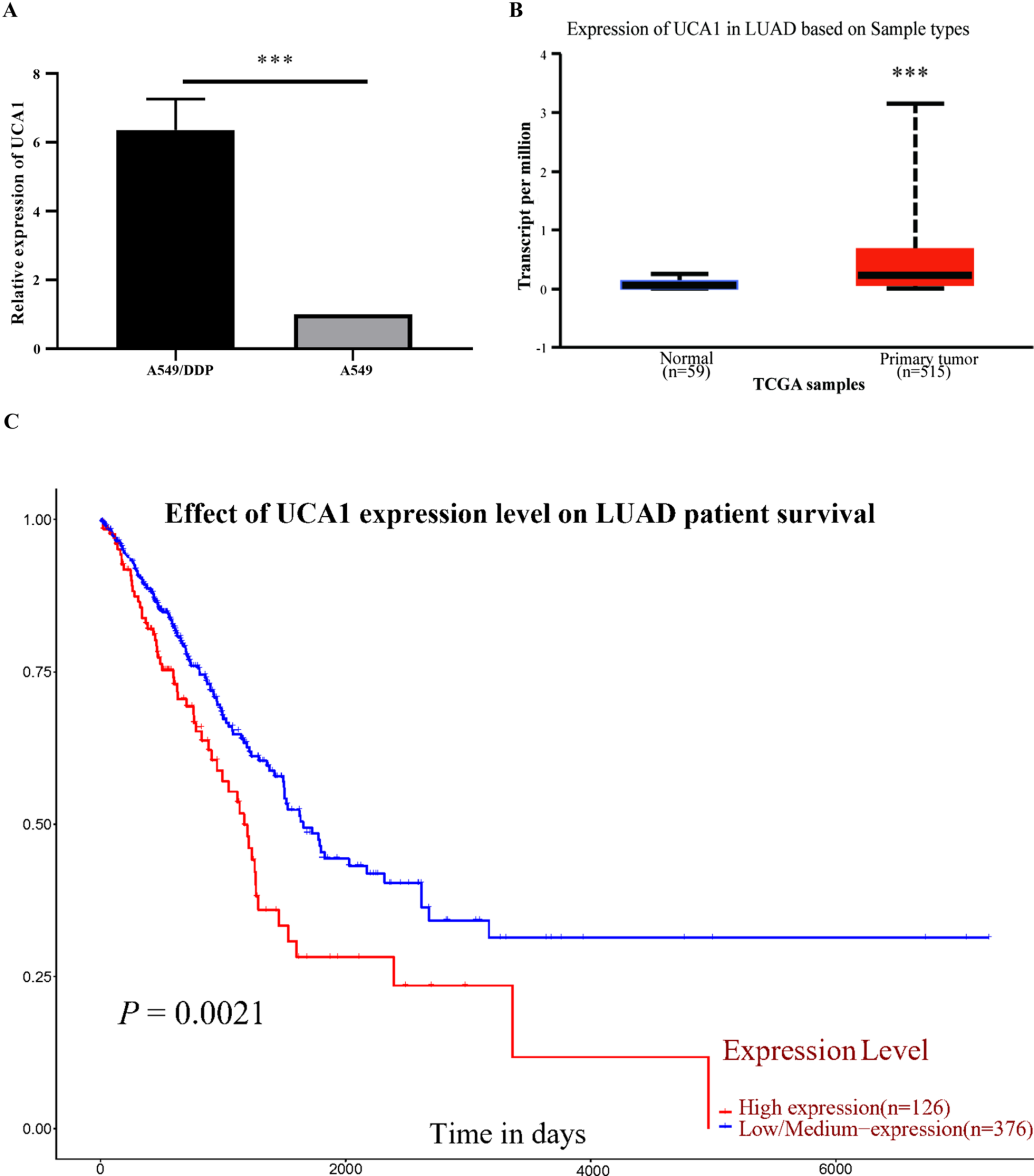
Expression of UCA1 in LUAD and LUAD cell lines and its role in poor prognosis of patients with LUAD. **A** Expression level of UCA1 was significantly higher in A549/DDP cells than in A549 cells. **B** UCA1 was highly expressed in LUAD cancer tissues than in corresponding NT examined by RT-qPCR. **C** TCGA database indicated that UCA1 expression was higher in tumor tissues than in adjacent normal tissues. **D** Kaplan Meier analysis showed that patients with LUAD having higher UCA1 expression had a shorter survival time. *NT* normal tissue; *P < 0.05, **P < 0.01, ***P < 0.001

In a previous study, we screened the lncRNAs between A549/DDP cells and A549 cells through a high-throughput microarray. UCA1 was one of the significantly upregulated genes in A549/DDP cells. To further confirm this result, we detected UCA1 expression in A549 and A549/DDP cells, and found that UCA1 was highly expressed in A549/DDP cells (*t* = 10.35, *P* < 0.001, Fig. 1A), which was consistent with previous findings. Next, we investigated the UCA1 expression levels in LUAD tissues and normal adjacent tissues through the TCGA database and found that the expression of UCA1 was higher in cancer tissues than in adjacent tissues (http://ualcan.path.uab.edu/cgibin/TCGAExResultNew2.pl?genenam=UCA1&ctype=LUAD, *P* < 0.001, Fig. 1B).

In addition, the 433 patients from the TCGA database were divided into 2 groups according to gene expression to investigate the correlation between UCA1 expression and clinical characteristics of patients with LUAD. Patients with the expression levels higher than the median were classified into the high-expression group; otherwise, they were classified into the low-expression group. The result showed no correlations between UCA1 expression and age, sex, T stage, N stage, smoking history, and tumor location. UCA1 was significantly associated with the M stage (*P* = 0.019) and clinical stage (*P* = 0.046, Table 2). The Kaplan–Meier analysis showed that the overall survival time of patients with high UCA1 expression levels was significantly shorter than those with low UCA1 expression levels (http://ualcan.path.uab.edu/cgi-bin/TCGA-survival1.pl?genenam=UCA1&ctype=LUAD, *P* = 0.021, Fig. 1C).

**Table 2:** Relationship between expression of UCA1 expression and clinicopathological characteristics in patients with LUAD. N(%)

Group	Total (n = 433)	UCA1		*P* value
		High expression	Low expression	
Gender				0.229
Male	198	93 (46.97)	105 (53.03)	
Female	235	124 (52.77)	111 (47.23)	
Age (year)				0.598
> 65	224	115 (51.34)	109 (48.66)	
≤ 65	209	102 (48.80)	107 (51.20)	
N stage				
N0	281	142 (50.53)	139 (49.47)	0.706
N1–3	142	69 (48.59)	73 (51.41)	
NX^a^	10	6 (60.00)	4 (40.00)	
T stage				
T1–2	376	185 (49.20)	191 (50.80)	0.383
T3–4	54	30 (55.56)	24 (44.44)	
TX^a^	3	2 (66.67)	1 (33.33)	
M stage				
M0	286	137 (47.90)	149 (52.10)	0.019*
M1	20	15 (75.00)	5 (25.00)	
MX^a^	127	65 (51.18)	62 (48.82)	
Clinical stage				0.046*
I, II	344	164 (47.67)	180 (52.32)	
III, IV	89	53 (59.55)	36 (40.45)	
Smoking				0.434
No	66	36 (54.55)	30 (45.45)	
Yes	277	181 (49.32)	186 (50.68)	
Tumor location				0.217
Superior lobe of left lung	108	61 (56.48)	47 (43.52)	
Inferior lobe of left lung	65	28 (43.08)	37 (56.92)	
Superior lobe of right lung	159	72 (45.28)	87 (54.72)	
Middle lobe of right lung	17	10 (58.82)	7 (41.18)	
Inferior lobe of right lung	84	46 (54.76)	38 (45.23)	

^a^“X” means that the tumor could not be evaluated or measured. The clinicopathological data of these patients was not included in statistical tests

*The difference was statistically significant

These results showed the role of UCA1 in LUAD cancer development and cisplatin resistance and its potential as a biomarker to predict poor prognosis and cisplatin resistance in patients with LUAD.

### UCA1 promoted cell proliferation and reduced sensitivity to cisplatin in the LUAD cell lines

**Fig. 2:**
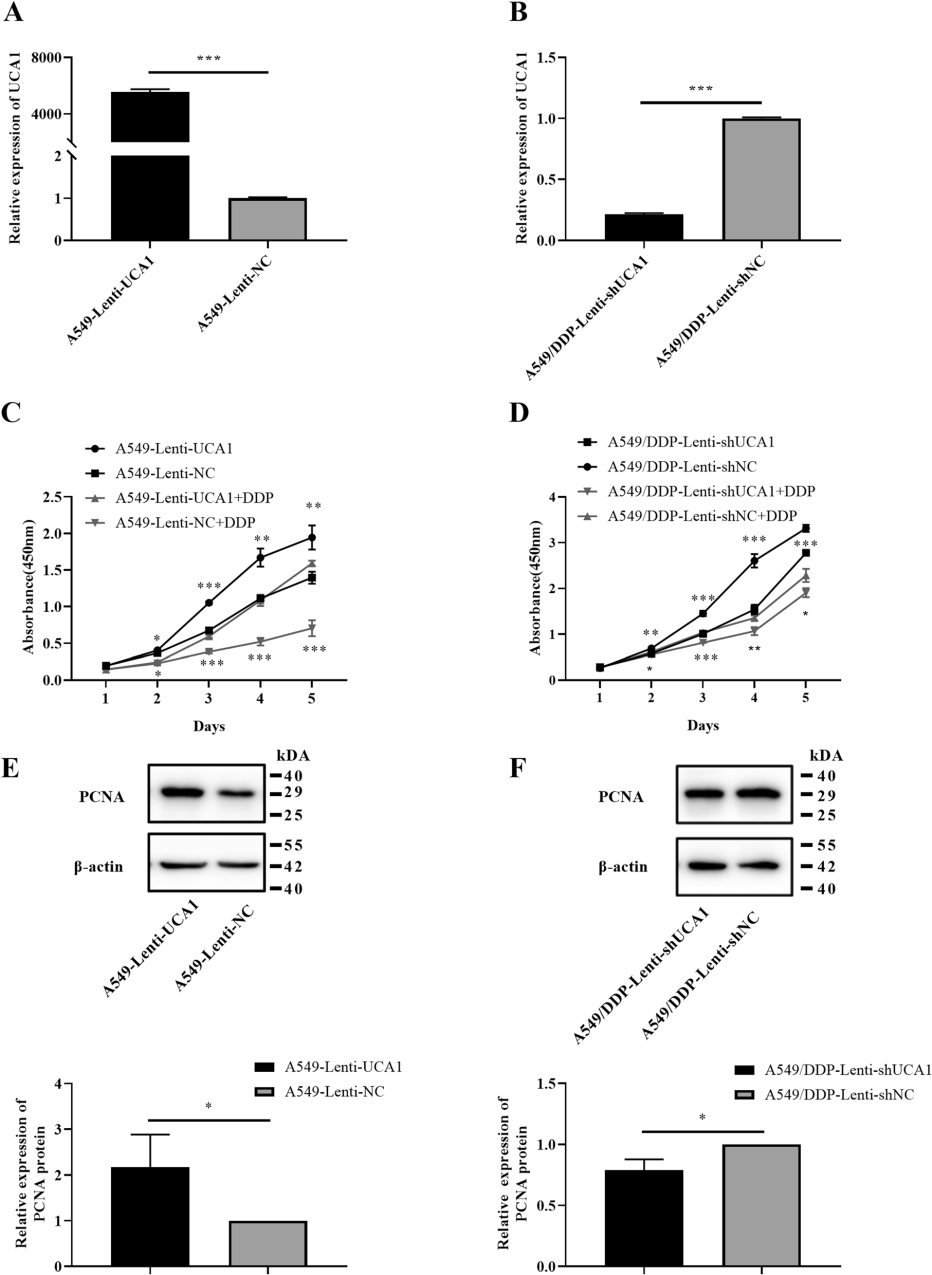
UCA1 contributed to the proliferative capacity of LUAD cells. **A**, **B** Lentivirus-mediated UCA1 overexpression and knockdown in A549 cells and A549/DDP cells were examined by RT-qPCR. **C**, **D** Growth curves of cells were evaluated by CCK-8 assays after overexpressing and knocking down UCA1 in A549 cells and A549/DDP cells. **E**, **F** Protein expression levels of PCNA in A549 cells and A549/DDP cells were determined by Western blot analysis, which indicated that UCA1 promoted LUAD cell proliferation. *P < 0.05, **P < 0.01, ***P < 0.001

RT-PCR showed that the RNA expression of UCA1 was markedly elevated in the overexpression group A549-Lenti-UCA1 compared with the negative control group A549-Lenti-NC (*t* = 54.71, *P* < 0.001, Fig. 2A). The UCA1 expression level decreased significantly in the A549/DDP-Lenti-shUCA1 group than in the A549/DDP-Lenti-shNC group (*t* = 95.10, *P* < 0.001, Fig. 2B). The cells with stable UCA1 overexpression and knockdown were established.

Next, we evaluated the role of UCA1 in LUAD cell proliferation. It was shown that the absorbance at 450 nm was higher in the A549-Lenti-UCA1 group than in the A549-Lenti-NC group after 24 h, 48 h, 72 h, 96 h, and 120 h (all *P* values < 0.05) after UCA1 overexpression (Fig. 2C). Under the effect of 2 μg/mL cisplatin, the absorbance values were still higher in the A549-Lenti-UCA1 group than in the A549-Lenti-NC group (all *P* value < 0.05, Fig. 2C). In contrast, the absorbance at 450 nm after 48 h, 72 h, 96 h and 120 h (all *P* values < 0.05) was lower in the A549/DDP-Lenti-shUCA1 group than in the A549/DDP-Lenti-shNC group (Fig. 2D). After 4 μg/mL cisplatin treatment, except for 24 h, the proliferation levels of the A549/DDP-Lenti-shNC cell lines were still stronger than that of UCA1 knockdown cell lines (all *P* values < 0.05, Fig. 2D). PCNA was an auxiliary protein involved in DNA replication and has been confirmed to be an indicator to evaluate the proliferation status of tumor cells [23, 24]. PCNA protein expression significantly increased after UCA1 overexpression (*t* = 2.819, *P* = 0.0479, Fig. 2E) and decreased after UCA1 knockdown (*t* = 4.136, *P* = 0.0144, Fig. 2F). These results demonstrated that UCA1 could promote the proliferation of LUAD cells and reduce sensitivity to cisplatin in LUAD cell lines.

### UCA1 promoted the migration and invasion of LUAD cells

**Fig. 3:**
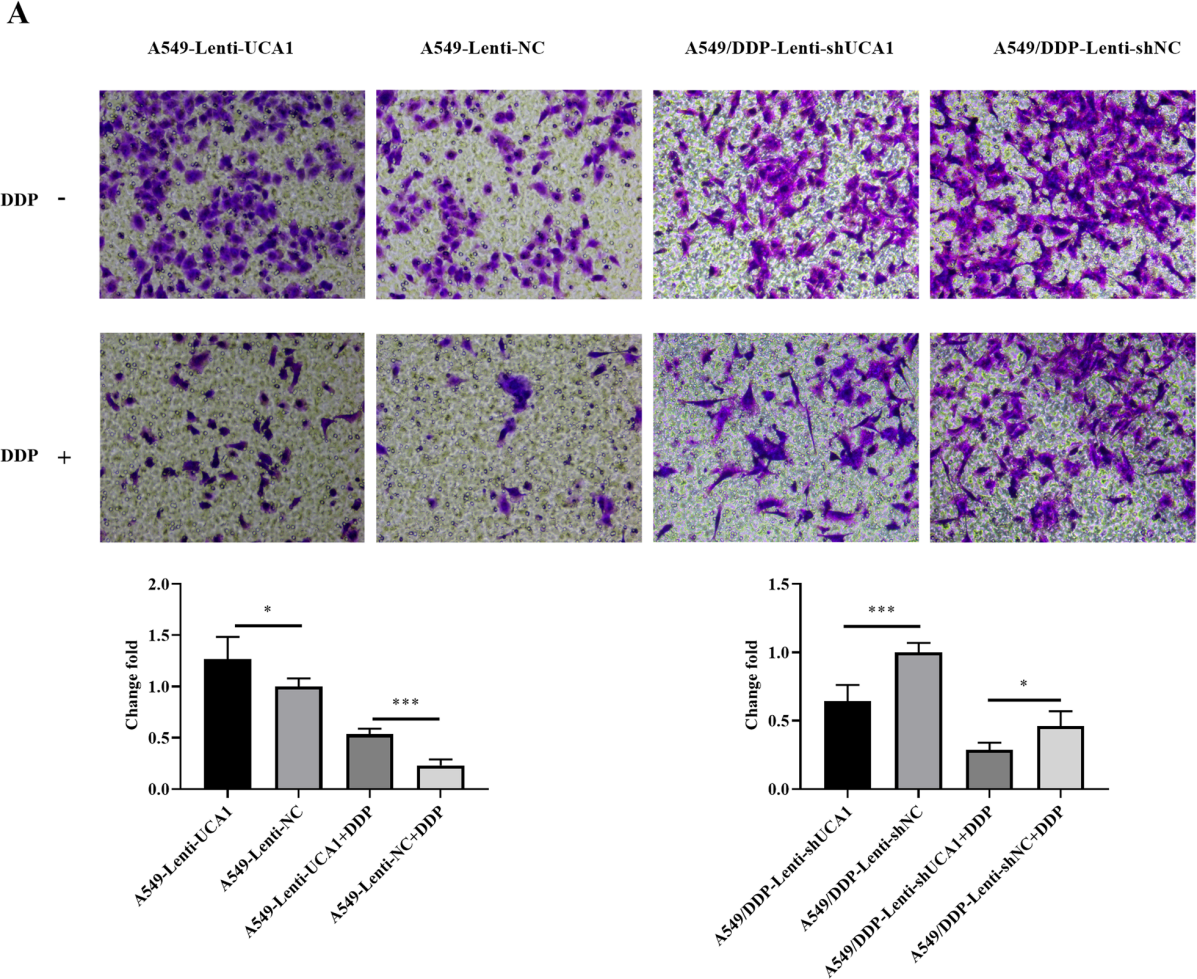

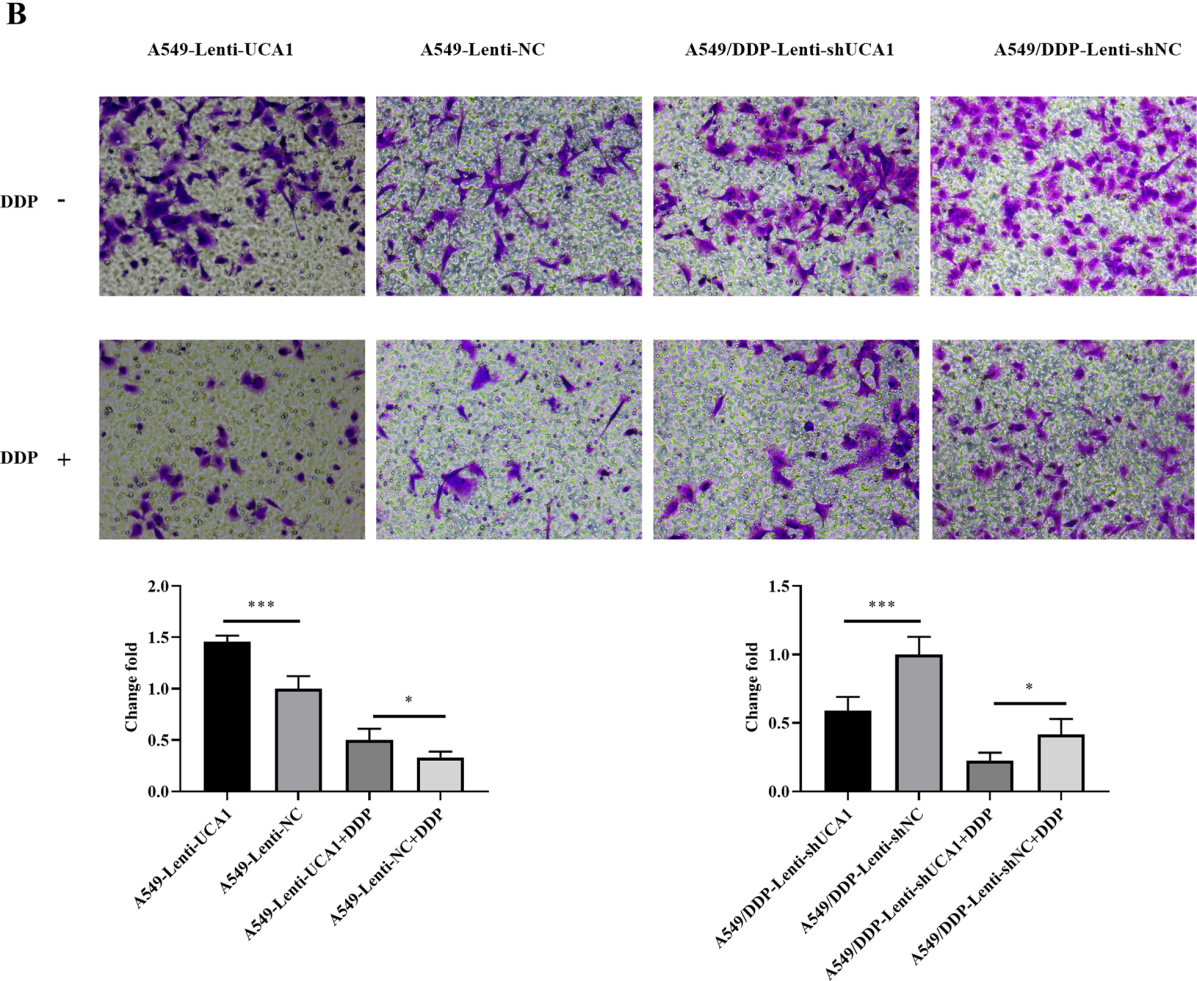
UCA1 promoted LUAD cell invasion and migration. **A**, **B** Cell migration was determined by Transwell assays after UCA1 overexpression and knockdown in A549 cells and A549/DDP cells. **C**, **D** Cell invasion was detected by Transwell assays with Matrigel after UCA1 overexpression and knockdown. *P < 0.05, **P < 0.01, ***P < 0.001

We found that the number of A549-Lenti-UCA1 migrating cells passing through the Transwell chamber was significantly higher than that in the control group (*t* = 2.596, *P* = 0.0318, Fig. 3A). After treatment with 2 μg/mL cisplatin, this trend was still obvious (*t* = 8.352, *P* < 0.0001, Fig. 3A). On the contrary, the A549/DDP-Lenti-shUCA1 group had fewer migrating cells than the A549/DDP-Lenti-shNC group (*t* = 5.754, *P* = 0.0004, Fig. 3A). The knockdown cells were treated with 4 μg/mL cisplatin, and the result was the same as that without the cisplatin treatment group (*t* = 3.307, *P* = 0.0107, Fig. 3A).

The results of the Matrigel invasion assays also showed that UCA1 significantly enhanced the cell invasion capability in A549 cells (*t* = 7.537, *P* < 0.0001; *t* = 3.173, *P* = 0.0131, Fig. 3B). However, the results were reversed after the knockdown of UCA1 (*t* = 5.568, *P* = 0.0005; *t* = 3.325, *P* = 0.0105, Fig. 3B). Migration and invasion assays showed that stable UCA1 overexpression significantly promoted the A549 cell migration and invasion ability, whereas UCA1 knockdown reduced the A549/DDP cell migration and invasion ability, whether under the influence of cisplatin.

### UCA1 enhanced the clonogenic capability of LUAD cells

**Fig. 4:**
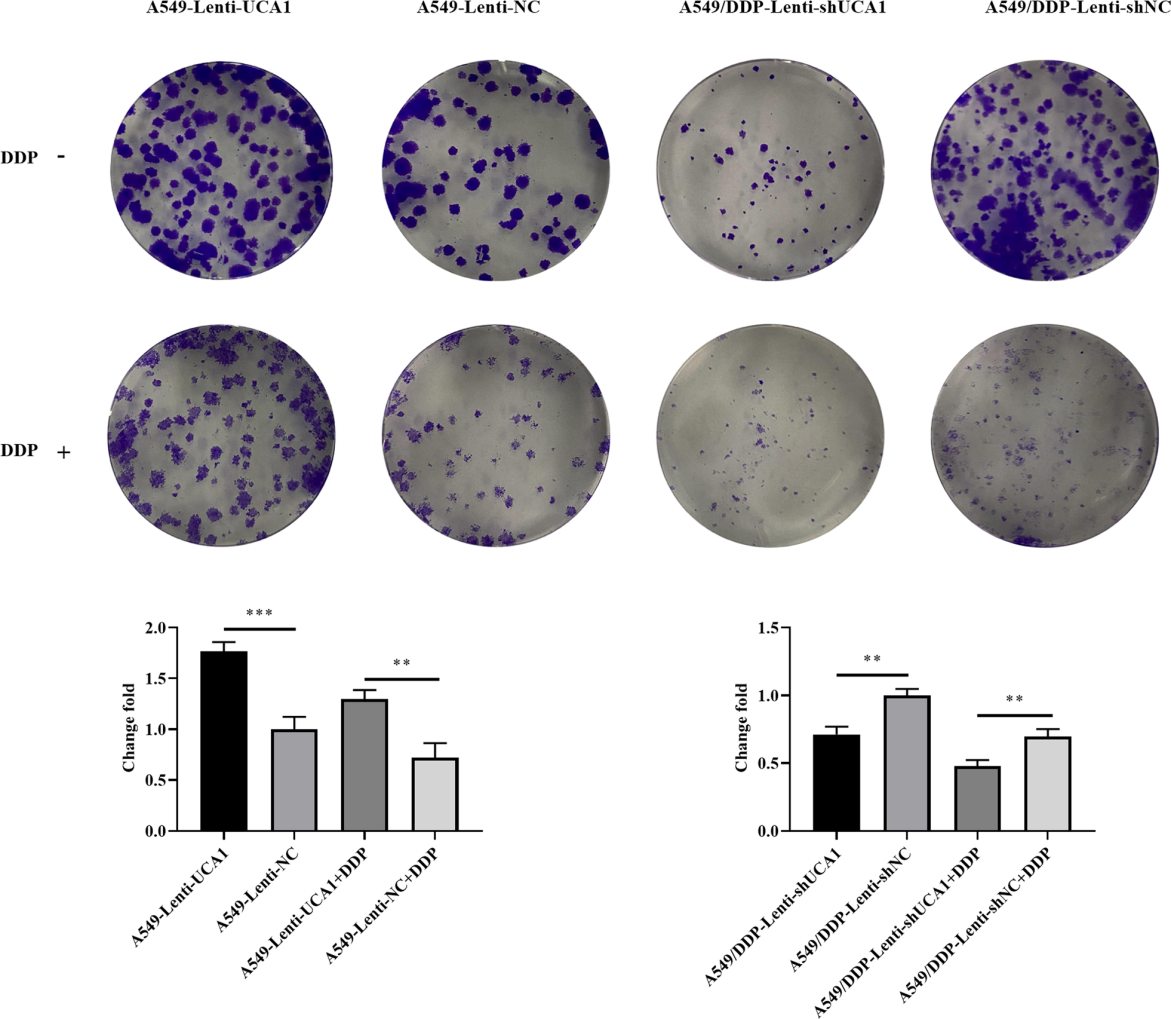
UCA1 enhanced LUAD cell clonogenic ability. **A**, **B** Colony-forming assays showed significantly more colonies after UCA1 overexpression in A549 cells compared with negative control, and UCA1 knockdown reversed the increase in cloning ability. *P < 0.05, **P < 0.01, ***P < 0.001

The UCA1 overexpression group possessed a higher colony-forming ability than the NC group (*t* = 8.766, *P* = 0.0009; *t* = 5.935, *P* = 0.0040; Fig. 4A). This result was reversed in the UCA1 knockdown group (*t* = 6.649, *P* = 0.0027; *t* = 5.375, *P* = 0.0058; Fig. 4A). These results demonstrated that UCA1 markedly enhanced the colony-forming ability of LUAD cells.

### UCA1 enhanced cisplatin resistance of LUAD cells by ERCC1

**Fig. 5:**
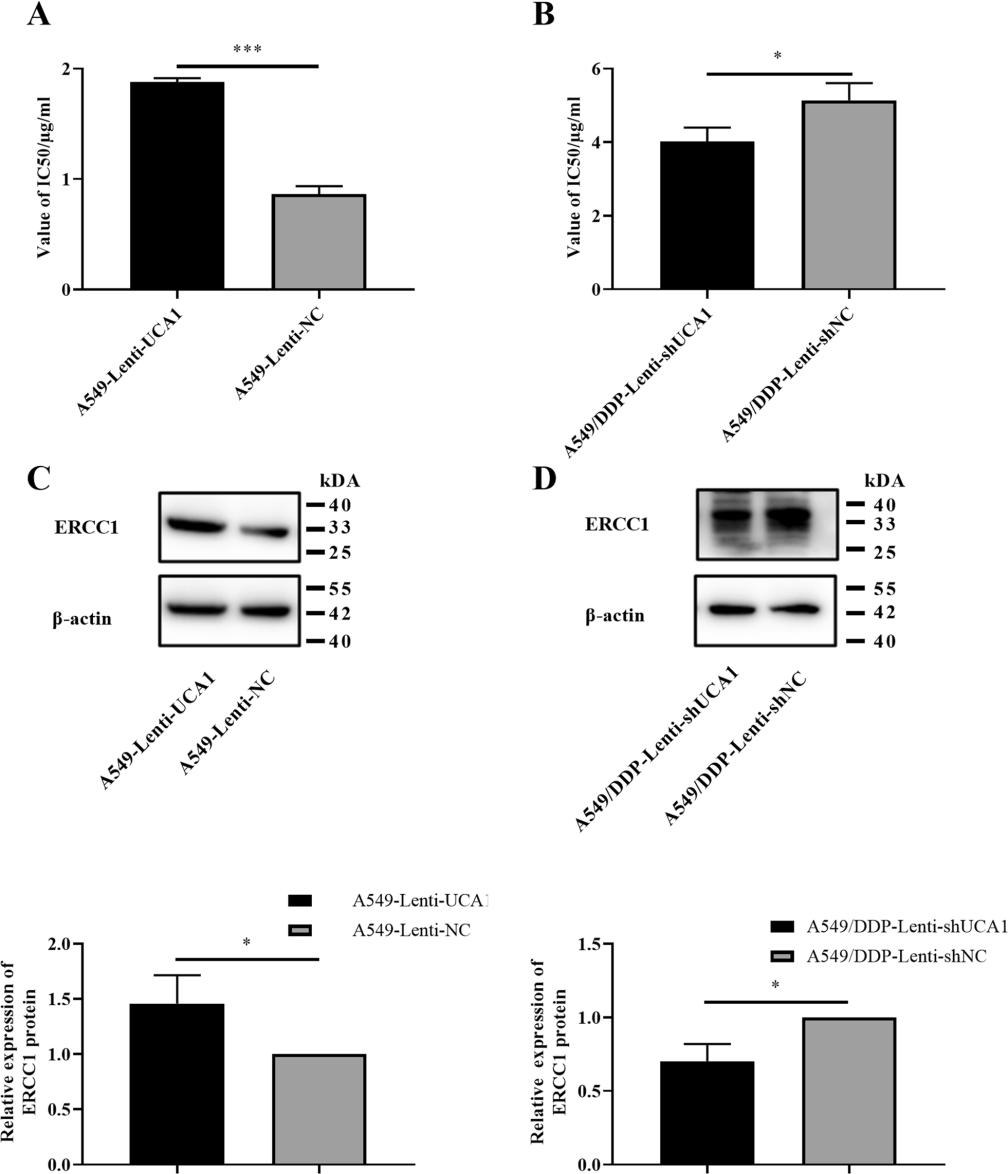
UCA1 enhanced the cisplatin resistance ability of LUAD cell lines. **A** UCA1-overexpressing A549 cells were more resistant to cisplatin, with higher IC50. **B** UCA1-knockdown A549/DDP cells were more sensitive to cisplatin, with lower IC50. **C** Western blot analysis of ERCC1 protein showed that UCA1 could enhance the cisplatin resistance of A549 cells. **D** UCA1 knockdown reduced the protein expression level of ERCC1. *P < 0.05, **P < 0.01, ***P < 0.001

The cell IC50 assay showed that the IC50 value of cisplatin of A549 cells increased from 0.865 ± 0.071 μg/mL to 1.878 ± 0.037 μg/mL (*t* = 21.92, *P* < 0.001, Fig. 5A). After UCA1 knockdown, the IC50 value of A549/DDP cells decreased from 5.135 ± 0.472 μg/mL to 4.021 ± 0.377 μg/mL (*t* = 3.193, *P* = 0.0331, Fig. 5B).

ERCC1 protein has been confirmed to be highly correlated with cisplatin resistance in NSCLC [25]. We detected ERCC1 expression in the cells and found that ERCC1 expression increased with the increase in UCA1 expression (*t* = 3.084, *P* = 0.0368, Fig. 5C) and decreased with the decrease in UCA1 expression (*t* = 4.393, *P* = 0.0118, Fig. 5D). Based on the results, as ERCC1 was involved in the DNA repair pathway, we inferred that UCA1 might activate the DNA repair pathway to enhance cisplatin resistance of LUAD cells.

### UCA1 promoted proliferation and cisplatin resistance of LUAD cells in vivo

**Fig. 6:**
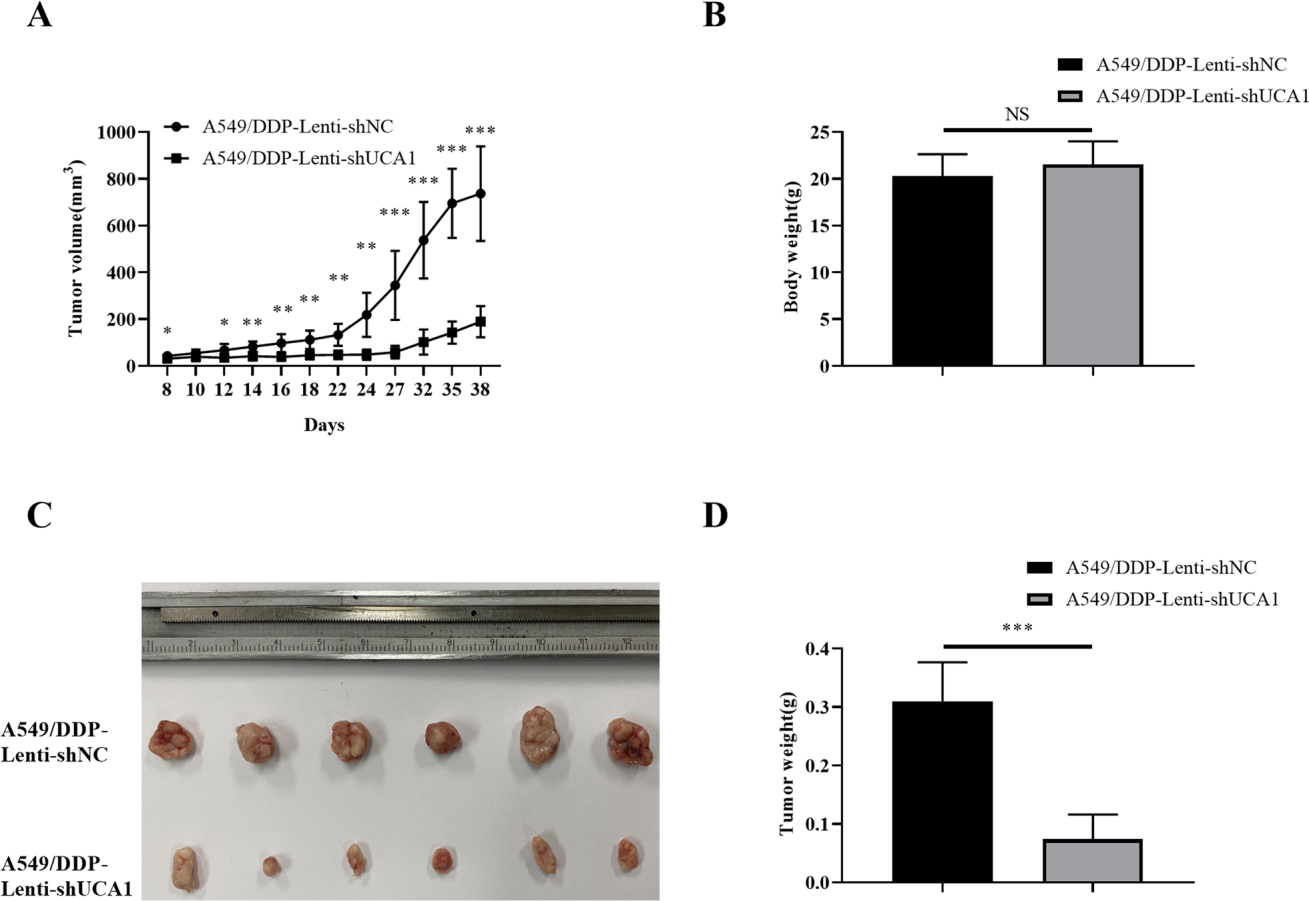
UCA1 promoted the growth and cisplatin resistance in A549/DDP cells in vivo*.* **A** Growth rate of the transplanted tumor was significantly slower in the UCA1 knockdown group than in the control group. **B** Average weight of nude mice in the UCA1 knockdown group and control group showed no significant difference between groups. **C** Volume of the transplanted tumor was significantly smaller in the A549/DDP-Lenti-shUCA1 group than in the control group; **D** weight of transplanted tumor was significantly higher in the A549/DDP-Lenti-shNC group than in the UCA1 knockdown group. NS means no statistically difference. *NS* not significant; *P < 0.05, **P < 0.01, ***P < 0.001

Tumor xenograft models were employed to further evaluate the effect of UCA1 on tumor growth and cisplatin resistance in vivo. As shown in Fig. 6A, the transplanted xenograft tumors were constructed after 8 days. No significant difference was found in the volume of transplanted tumors except on the 10th day. At other time points, the tumor volume was significantly higher in the A549/DDP-Lenti-shNC group than in the A549/DDP-Lenti-shUCA1 group (all* P* values < 0.05). The weight of nude mice was slightly lower in the A549/DDP-Lenti-shNC group (20.300 ± 2.326 g) than in the A549/DDP-Lenti-shUCA1 group (21.533 ± 2.482 g), but the difference was not statistically significant (Fig. 6B). However, the weight of transplanted tumor was significantly lower in the A549/DDP-Lenti-shUCA1 group (0.074 ± 0.042 g) than in the A549/DDP-Lenti-shNC group (0.310 ± 0.066 g) (*t* = 7.354, *P* < 0.001, Fig. 6C, D). The tumor growth was most significantly inhibited in mice following UCA1 knockdown compared with that in the NC groups, indicating that UCA1 played an important role in regulating the growth of LUAD cells in vivo.

**Fig. 7:**
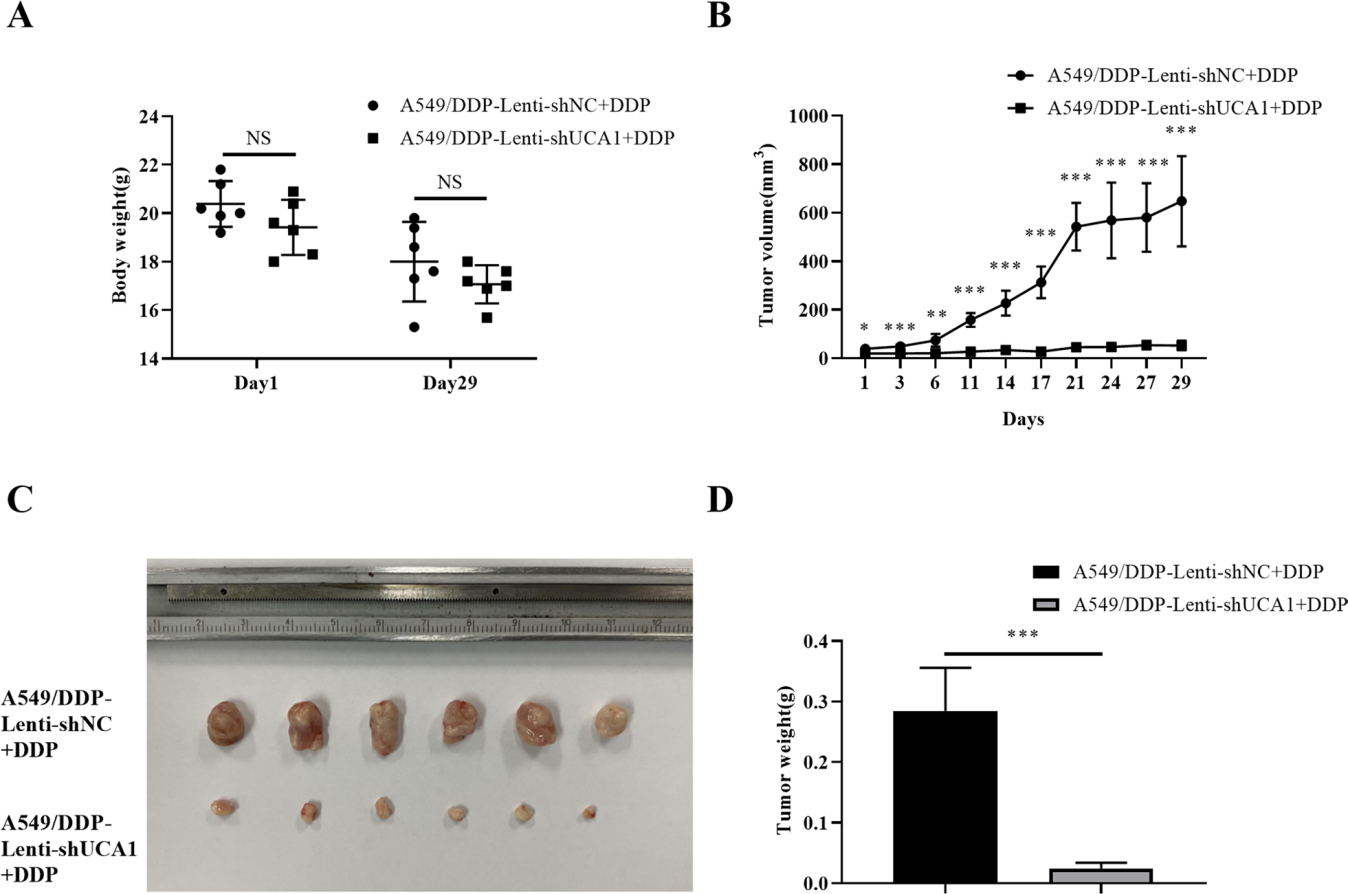
Knockdown with UCA1 restored cisplatin sensitivity of cisplatin-resistant cell lines in vivo. **A** Two groups of nude mice treated with cisplatin showed different degrees of weight loss. **B** Growth of the transplanted tumor in the UCA1 knockdown group was significantly inhibited by cisplatin. **C** Transplanted tumor in the A549/DDP-Lenti-shNC + DDP and A549/DDP-Lenti-shUCA1 + DDP groups. (D) Weight of the transplanted tumor was significantly lower in the A549/DDP-Lenti-shUCA1 + DDP group than in the A549/DDP-Lenti-shNC + DDP group. *NS* not significant. *P < 0.05, **P < 0.01, ***P < 0.001

With cisplatin treatment, the bodyweight in the A549/DDP-Lenti-shUCA1 + DDP group decreased from 19.417 ± 1.137 g to 17.067 ± 0.784 g and that in the A549/DDP-Lenti-shNC + DDP group decreased from 20.383 ± 0.947 g to 18.000 ± 1.643 g, with no significant difference between the two groups both before and after chemotherapy (Fig. 7A). After an intraperitoneal injection of cisplatin, the inhibition degree of the transplanted tumor was significantly higher in the A549/DDP-Lenti-shUCA1 + DDP group than in the control group. During chemotherapy, the tumor volume was significantly lower in the A549/DDP-Lenti-shUCA1 + DDP group than in of the A549/DDP-Lenti-shNC + DDP group (all* P* values < 0.05, Fig. 7B). After chemotherapy, the tumor weight in the A549/DDP-Lenti-shUCA1 + DDP group was 0.025 ± 0.009 g, which was significantly lower than that in the A549/DDP-Lenti-shNC + DDP group (0.285 ± 0.071 g, *t* = 0.885, *P* < 0.001, Fig. 7C, D). These results demonstrated that the knockdown with UCA1 restored the sensitivity of A549/DDP cells to cisplatin. Taken together, UCA1 promoted proliferation and cisplatin resistance of LUAD cells in vivo.

### RNA-binding protein ENO1 of UCA1

**Fig. 8:**
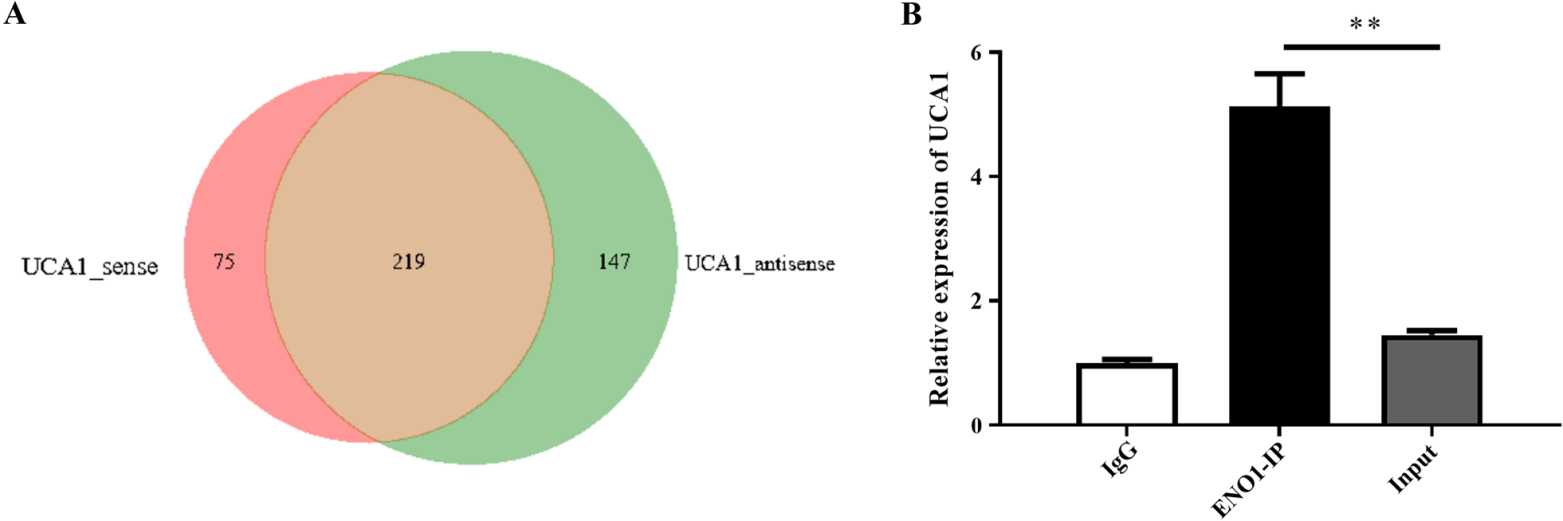
RNA-binding protein ENO1 of UCA1 was obtained. **A** A Wayne diagram showed the proteins of UCA1_sense and UCA1_antisense detected by protein MS after RNA pulldown. **B** RIP assays were applied using anti-ENO1 antibodies and anti-IgG antibodies with extractions from A549 cells, and the UCA1 expression levels in the immunoprecipitates were detected by RT-qPCR assays. *P < 0.05, **P < 0.01, ***P < 0.001

After RNA pulldown, the products of UCA1_sense and UCA1_antisense were visualized by silver staining (Additional file 1: Fig. S1). And through protein mass spectrometry (MS), a total of 441 proteins were identified, among which 219 were identified in 2 samples, while 75 proteins were identified uniquely in UCA1_sense and 147 unique proteins in UCA1_antisense (Fig. 8A). Among these, some proteins related to tumor chemotherapy resistance were summarized in Table 3.

**Table 3:** Detailed information of the proteins related to drug resistance of UCA1_sense and UCA1_antisense detected by protein MS after RNA pulldown

Identified by	Protein ID	Function	Ref.
Both UCA1_sense and UCA1_antisense	sp|P04406|G3P_HUMAN	Inhibit cytarabine resistance of A549 cells	[26]
	sp|P07437|TBB5_HUMAN	Be linked to paclitaxel chemoresistance of ovarian cancer cells	[27]
	sp|P25705|ATPA_HUMAN	Lead to 5-FU resistance of colon cancer cells	[28]
	sp|P60174|TPIS_HUMAN	Be an anti-drug resistance agent in gastric cancer	[29]
	sp|P00338|LDHA_HUMAN	serve an important role in docetaxel resistance in castration‑resistant prostate cancer	[30]
	sp|P07737|PROF1_HUMAN	Synergize with chemotherapeutic drugs to induce tumor cell death	[31]
	sp|P31943|HNRH1_HUMAN	Result in acquired chemoresistance to thymidine phosphorylase activated fluoropyrimidine anticancer drugs	[32]
	sp|O43175|SERA_HUMAN	Be a critical driver for sorafenib resistance in hepatocellular carcinoma	[33]
	sp|P15924|DESP_HUMAN	Increase the sensitivity to anticancer drug-induced apoptosis in NSCLC	[34]
	sp|P04792|HSPB1_HUMAN	Inhibit cell apoptosis induced by paclitaxel in ovarian cancer	[35]
	sp|P06733|ENOA_HUMAN	Be a partner protein of fibroblast growth factor receptor‐like 1 promoting chemoresistance of small‐cell lung cancer	[36]
UCA1_sense	sp|Q8N163|CCAR2_HUMAN	Promote drug resistance of colonospheres colon cancer cells	[37]
	sp|P11908|PRPS2_HUMAN	Be a target gene regulated by miRNAs which co-regulate vincristine resistance in childhood acute lymphoblastic leukemia	[38]
	sp|Q13045|FLII_HUMAN	Silencing of FLI1 sensitizes the resistant cells to BRAF inhibitors in melanoma cells	[39]
	sp|Q9Y6E2|BZW2_HUMAN	Promote rapamycin resistance of hepatocellular carcinoma cells	[40]
	sp|P60981|DEST_HUMAN	Be a differentially expressed protein related to platinum resistance in ovarian cancer	[41]
	sp|Q05639|EF1A2_HUMAN	Inhibit chemotherapy-induced apoptosis resulting in chemoresistance	[42]
	sp|Q92769|HDAC2_HUMAN	BE a target to overcome therapeutic resistance of adenocarcinoma against chemotherapeutics in pancreatic cancer	[43]
	sp|P51991|ROA3_HUMAN	Be upregulated in cisplatin-resistant breast cancer cells	[44]
	sp|P33993|MCM7_HUMAN	Be inhibited by simvastatin to suppress tamoxifen-resistant breast cancer cells growth	[45]
	sp|P14174|MIF_HUMAN	As a therapeutic target for overcoming resistance to proteasome inhibitors in myeloma	[46]
UCA1_antisense	sp|P80723|BASP1_HUMAN	Interact with oestrogen receptor α and modify the tamoxifen response in breast cancer	[47]
	sp|P14866|HNRPL_HUMAN	Knockdown of HNRPL enhanced the level of DNA breakage and sensitivity of colorectal cancer cells to oxaliplatin	[48]
	sp|Q15019|SEPT2_HUMAN	LncRNA FGD5-AS1/miR-497-5p/SEPT2 axis increased cisplatin-resistance in laryngeal squamous cell carcinoma	[49]
	sp|Q15717|ELAV1_HUMAN	Contribute to TRAIL resistance by restricting death receptor 4 expression in pancreatic cancer	[50]
	sp|P13797|PLST_HUMAN	Be a critical regulator of paclitaxel sensitivity of MDA-MB-231 cells	[51]
	sp|P21333|FLNA_HUMAN	Modulate chemosensitivity to docetaxel in triple-negative breast cancer through the MAPK/ERK pathway	[52]
	sp|P06493|CDK1_HUMAN	Cooperate with UBE2C to promote cisplatin resistance in ovarian cancer	[53]
	sp|P47929|LEG7_HUMAN	Be a potential predictive marker of chemotherapy resistance in oral squamous cell carcinoma	[54]
	sp|Q8TEX9|IPO4_HUMAN	Enhance cervical cancer cisplatin sensitivity	[55]
	sp|Q9P258|RCC2_HUMAN	Block spontaneous- or staurosporine-induced apoptosis in three cancer cell lines	[56]

Enolase 1 (ENO1), known as coding enolization enzyme 1, played a key role in glucose metabolism and tumor development [57]. ENO1 was highly expressed in lung cancer tissues and promoted LUAD progression by regulating the glycolytic pathway [58]. We explored by the RIP experiment whether ENO1 was bound to UCA1. The results confirmed that UCA1 was highly expressed in immunoprecipitated ENO1 (*t* = 6.859, *P* = 0.0024, Fig. 8B), indicating that ENO1 possibly was one of the RNA-binding proteins (RBP) of UCA1. It was quite possible that UCA1 might co-engage with ENO1 in regulating cisplatin resistance mechanisms in LUAD.

## Discussion

Using a lncRNA microarray, we analyzed the differentially expressed genes between cisplatin-sensitive cell line A549 and cisplatin-resistant cell line A549/DDP, in which UCA1 expression was upregulated. Consistent with the results from the TCGA database, we discovered by RTqPCR that UCA1 in LUAD tissues and A549/DDP cells were highly expressed. UCA1 expression had a correlation with metastasis, worse clinical staging, and prognosis, and was consistent with previous findings [59–61]. The results suggested that UCA1 was involved in the development and cisplatin resistance of LUAD and could be used as a biomarker to predict the prognosis and cisplatin resistance.

We found that the capability of cell proliferation, migration, and invasion was enhanced after UCA1 overexpression. The existing research results showed that UCA1 participated in cell proliferation [61], migration, and invasion [62, 63] to regulate tumor progression. Therefore, we detected the IC50 value of LUAD cell lines and confirmed that the IC50 value to cisplatin increased after UCA1 overexpression. This suggested that UCA1 was likely to increase the cisplatin resistance ability of LUAD. Nevertheless, the specific mechanism still needs further research and remains to be explored.

RBP could bind to their target RNA, form ribonucleoprotein complexes, and regulate gene expression after transcription [64]. A total of 441 proteins were identified. We selected the ENO1 protein to perform RIP experiments. The RIP result showed that UCA1 expression increased in the ENO1 immunoprecipitation complex, indicating that ENO1 was likely to be the RBP of UCA1. ENO1 has been identified to be involved in the process of drug resistance in many types of tumor cells [36, 65, 66]. Therefore, UCA1 may bind with ENO1 and affect ENO1 expression and its target genes.

## Conclusions

In summary, UCA1 was involved in the regulation of cell proliferation, migration, invasion, and enhanced cell cisplatin resistance in LUAD. High UCA1 expression predicted a poor prognosis and was associated with distant metastasis and high tumor grade in patients with LUAD. ENO1 is an RNA-binding protein of UCA1. This study provided a potential diagnostic marker and therapeutic target for lung cancer and cisplatin resistance of LUAD, which was expected to effectively prolong the survival time and improve the quality of life of patients with LUAD.

## Supplementary Information

**Additional file 1: Figure S1.** The proteins of RNA pulldown were resolved through SDS-PAGE and visualized by silver staining.

**Additional file 2: Table S1.** The primers sequence of sense chain and antisense chain of LncRNA UCA1 for RNA pulldown.

## Data Availability

The datasets used and/or analyzed in the present study are available from the corresponding author on reasonable request.

## Abbreviations

CCK8: Cell count kit-8
CREB1: CAMP responsive element binding protein-1
DDP: Cisplatin
EMT: Epithelial–mesenchymal transition
ENO1: Enolase 1
ERCC1: Excision repair cross-complementing gene 1
FBS: Fetal bovine serum
FOSL2: Fos-like antigen 2
IC50: Half-maximal inhibitory concentration
lncRNA: Long noncoding RNA
LUAD: Lung adenocarcinoma
MS: Mass spectrometry
MOI: Multiplicity of infection
NC: Negative group
NRF2: Nuclear factor-erythroid 2-related factor 2
NSCLC: Non-small cell lung cancer
PBS: Phosphate buffer saline
PCNA: Proliferating cell nuclear antigen
RBP: RNA-binding proteins
RIP: RNA immunoprecipitation
RPMI-1640: Roswell Park Memorial Institute-1640
RT‑qPCR: Reverse transcription‑quantitative polymerase chain reaction
SCLC: Small-cell lung cancer
SPF: Specific pathogen-free
TCGA: The Cancer Genome Atlas
UCA1: Urothelial carcinoma-associated 1
WB: Western blotting
