# Supplementary figures and images for: Mechanistic study of lncRNA UCA1 promoting growth and cisplatin resistance in lung adenocarcinoma
Source: Cancer Cell Int. 2021 Sep 20;21:505. doi: 10.1186/s12935-021-02207-0 (PMC8454127; doi:10.1186/s12935-021-02207-0)

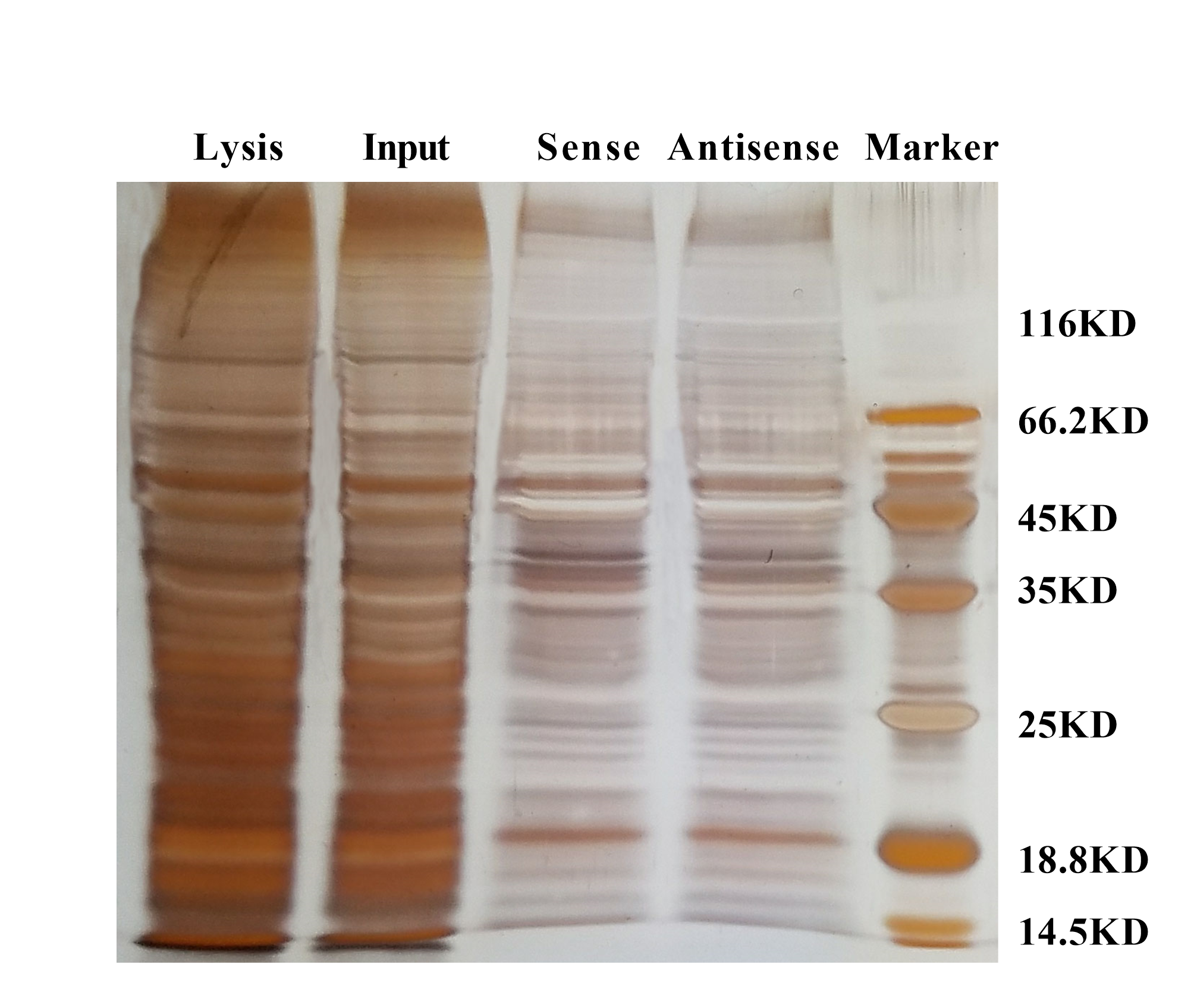

Supplement: Supplementary file 1 — Additional file 1: Figure S1. The proteins of RNA pulldown were resolved through SDS-PAGE and visualized by silver staining. [file 12935_2021_2207_MOESM1_ESM.tif]
