# Supplementary material for: Mechanistic study of lncRNA UCA1 promoting growth and cisplatin resistance in lung adenocarcinoma
Source: Cancer Cell Int. 2021 Sep 20;21:505. doi: 10.1186/s12935-021-02207-0 (PMC8454127; doi:10.1186/s12935-021-02207-0)
Supplement: Supplementary file 2 — Additional file 2: Table S1. The primers sequence of sense chain and antisense chain of LncRNA UCA1 for RNA pulldown. [file 12935_2021_2207_MOESM2_ESM.docx]

Supplementary Table 1. The primers sequence of sense chain and antisense chain of LncRNA UCA1 for RNA pulldown.

| UCA1 | Seq（5’-3’） | |
| --- | --- | --- |
|  | upstream primers | downstream primers |
| sense | TGACATTCTTCTGGACAATGAGTCCCATCA | ATCAGGCATATTAGCTTTAATGTAGGTGGC |
| antisense | ATCAGGCATATTAGCTTTAATGTAGGTGGC | TGACATTCTTCTGGACAATGAGTCCCATCA |
